# Oxygen-sensing neurons reciprocally regulate peripheral lipid metabolism via neuropeptide signaling in *Caenorhabditis elegans*

**DOI:** 10.1371/journal.pgen.1007305

**Published:** 2018-03-26

**Authors:** Rosalind Hussey, Nicole K. Littlejohn, Emily Witham, Erik Vanstrum, Jaleh Mesgarzadeh, Harkaranveer Ratanpal, Supriya Srinivasan

**Affiliations:** 1 Department of Molecular Medicine and Dorris Neuroscience Center, The Scripps Research Institute, La Jolla, CA, United States of America; 2 Department of Biology, University of California, San Diego, La Jolla, CA, United States of America; Baylor College of Medicine, UNITED STATES

## Abstract

The mechanisms by which the sensory environment influences metabolic homeostasis remains poorly understood. In this report, we show that oxygen, a potent environmental signal, is an important regulator of whole body lipid metabolism. *C*. *elegans* oxygen-sensing neurons reciprocally regulate peripheral lipid metabolism under normoxia in the following way: under high oxygen and food absence, URX sensory neurons are activated, and stimulate fat loss in the intestine, the major metabolic organ for *C*. *elegans*. Under lower oxygen conditions or when food is present, the BAG sensory neurons respond by repressing the resting properties of the URX neurons. A genetic screen to identify modulators of this effect led to the identification of a BAG-neuron-specific neuropeptide called FLP-17, whose cognate receptor EGL-6 functions in URX neurons. Thus, BAG sensory neurons counterbalance the metabolic effect of tonically active URX neurons via neuropeptide communication. The combined regulatory actions of these neurons serve to precisely tune the rate and extent of fat loss to the availability of food and oxygen, and provides an interesting example of the myriad mechanisms underlying homeostatic control.

## Author summary

We now appreciate that the sensory nervous systems of complex multicellular animals play a profound role in influencing energy balance, and body fat stores. Understanding the precise molecular mechanisms and neuroendocrine pathways by which the nervous system controls metabolic tissues has remained a tremendous challenge. Using the genetically tractable nematode *C*. *elegans*, we can dissect the critical sensory modalities that influence body fat storage and its mobilization, and their mechanisms of action. In this study, we identify the molecular mechanisms by which a salient environmental feature, oxygen, regulates the magnitude of fat loss via the counterbalancing actions of the BAG and URX oxygen sensory neurons.

## Introduction

The central nervous system plays a critical role in regulating whole body energy balance. In mammals, in addition to the role of the hypothalamus, there is good evidence showing that the sensory nervous system also regulates whole body metabolism [1–4]. However, the identification of discrete sensory modalities and the underlying signaling pathways that regulate metabolism in peripheral tissues has remained a tremendous challenge. As a result, basic questions regarding the molecular and physiological mechanisms underlying the neuronal control of lipid metabolism remain unknown even as rates of obesity, diabetes and their complications soar worldwide.

In the nematode *Caenorhabditis elegans*, many ancient mechanisms of sensory and metabolic regulation have been preserved, thus offering an opportunity to dissect the pathways by which the nervous system regulates lipid metabolism. The *C*. *elegans* nervous system is relatively well-defined both genetically and anatomically [5, 6], thus genes underlying discrete sensory modalities can be rapidly assessed for roles in whole body metabolism. The intestine is the seat of metabolic control and is the predominant depot for fat uptake, storage and mobilization. Thus, changes in whole body metabolism can be effectively encapsulated by monitoring metabolic readouts in the intestine. *C*. *elegans* sensory neurons play pivotal roles in regulating metabolism [7, 8]. At least 3 discrete sensory inputs: food availability [9–11], population density [12] and environmental oxygen [13] are relayed from chemosensory neurons to the intestine. These sensory inputs control the duration and magnitude of fat loss, effectively coupling environmental information with body fat metabolism via neuroendocrine hormones. The molecular and neuroendocrine mechanisms by which sensory information is relayed from the nervous system to the intestine has the tremendous potential to shed light on the interaction between genetic mechanisms and environmental conditions in controlling lipid metabolism.

Neuronal oxygen sensing is a sensory modality that is richly informative to *C*. *elegans*, which grow in environments replete with bacteria, their major food source. Respiring bacteria drop the local ambient concentration of oxygen from 21% (atmospheric) to a range between 10–13%. Worms presented with an oxygen gradient from 0–21% choose a range between 10–13%, and avoid higher and lower oxygen concentrations, the better to remain in areas that reflect the balance between sufficient food and oxygen [14, 15]. Importantly, these responses are distinct from hypoxia-related responses, which occur at 3% oxygen and below, are encoded by the conserved hypoxia genes including HIF-1, and are distinct from mechanisms of oxygen sensing during normoxia [16]. Oxygen preference within the normoxic range is encoded by two pairs of sensory neurons [17]. The URX neurons detect high (21%) oxygen via a guanylate cyclase called GCY-36, and initiate an aversive behavioral response [17, 18]. On the other hand, BAG neurons detect low (5–10%) oxygen via an inhibitory guanylate cyclase called GCY-33, and also initiate aversion [17, 19]. Thus, URX and BAG neurons function together to retain animals in food environments that contain an optimal balance of food and oxygen.

In a screen for neuronal regulators of fat metabolism, we uncovered a role for the URX neurons in regulating oxygen-dependent fat loss [13]. When fasted, body fat stores are metabolized for the production of energy. A time course of food withdrawal at atmospheric (21%) oxygen shows that the intestinal fat stores of *C*. *elegans* are steadily depleted, and by 3 hours young adults lose ~80% of their body fat stores; near-complete fat loss in the intestine occurs by 4 hours [13]. Wild-type animals exposed to 10% oxygen retain approximately twice as much body fat as those exposed to high oxygen. We previously showed that this effect is not regulated by changes in food intake or locomotor rates at high versus low oxygen [13]. Rather, it occurs because of a selective metabolic shift towards fat utilization that is differentially regulated by oxygen availability. In the intestine, this metabolic shift depends upon the conserved triglyceride lipase called ATGL-1, which is transcriptionally regulated by oxygen. Thus, animals exposed to high oxygen show greater fat loss via ATGL-1 activation, than those exposed to low oxygen for the same duration. This differential metabolic response to oxygen depends on neuronal oxygen sensing via the URX neurons, and functions via a sensor of molecular oxygen, the soluble guanylate cyclase GCY-36. We found that a gustducin-like Gα protein called GPA-8 functions as a negative regulator of GCY-36, and serves to limit the tonic, constitutive activity of URX neurons by repressing resting Ca^2+^ levels. Loss of GPA-8 disinhibits the tonic activity of the URX neurons at 10% oxygen (when they are normally silent), which leads to increased fat loss [13].

Our findings on the role of the URX neurons in regulating oxygen-dependent fat loss led us to contemplate a potential role for the BAG neurons, which reciprocally regulate behaviors associated with sensing low oxygen (5–10%) and more broadly, of the role of normoxic oxygen in regulating lipid metabolism [17, 19]. The URX and BAG neurons are activated at high and low oxygen, respectively [14, 15, 17, 19–21]. However, these neurons are known to be tonic sensors of oxygen, suggesting that they would need to be kept in the 'off state' by active repression. Neuronal mechanisms underlying repression of tonically active neurons are not well-defined. In this study, we report that BAG neurons play an important role in regulating oxygen-dependent fat loss. We find that BAG neurons relay sensory information about low oxygen to the URX neurons and inhibit them to maintain low tonic activity. The physiological consequence of this neuronal signaling is to reduce the rate and extent of fat loss in the intestine during low oxygen. Communication from BAG neurons to URX neurons occurs via the FLP-17 neuropeptide from the BAG neurons and its cognate receptor, the G protein coupled receptor EGL-6, which we show functions in the URX neurons. Thus, we find that BAG neurons play a critical role in modulating body fat homeostasis by controlling the resting properties of URX neurons. Our work points to a model in which neurons with opposing sensory roles establish a mutually reinforcing circuit via neuropeptide signaling, and function together to regulate metabolic homeostasis in the intestine.

## Results

### The soluble guanylate cyclase GCY-33 regulates intestinal lipid metabolism from the oxygen-sensing BAG neurons

**Fig 1:**
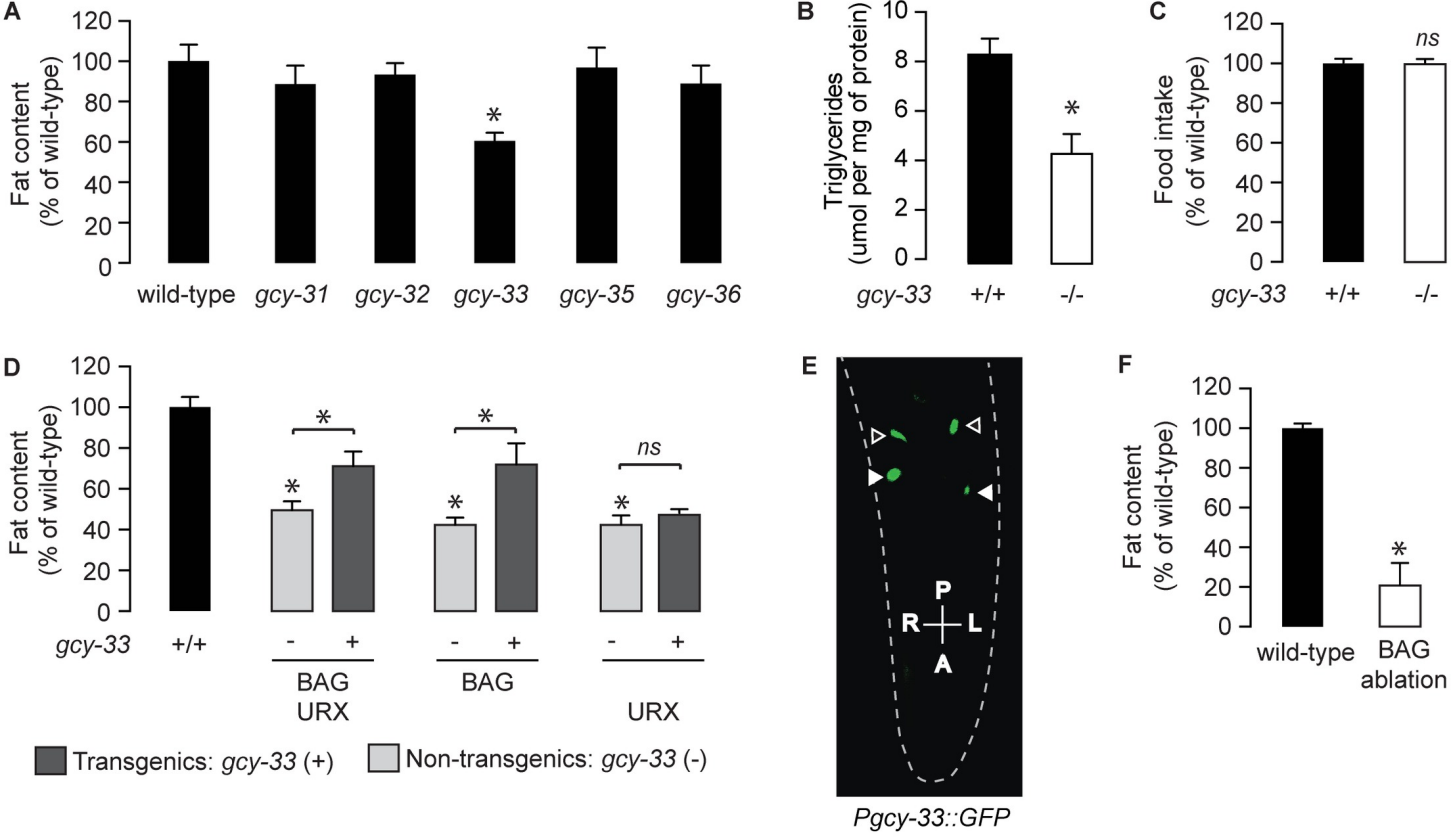
The oxygen sensor GCY-33 acts in BAG neurons to regulate lipid metabolism in intestinal cells. (A) Animals were fixed and stained with Oil Red O. Fat content was quantified for each genotype and is expressed as a percentage of wild-type animals + SEM (n = 12–20). *, p<0.05 by one-way ANOVA. See also S1 Fig. (B) Total triglyceride levels in wild-type and *gcy-33* mutants. Data are presented as μmol per mg of protein + SEM (n = 3). *, p<0.05 by Student’s t-test. (C) Food intake in *gcy-33* mutants expressed as a percentage of wild-type + SEM (n = 10). NS, not significant by Student’s t-test. (D) For each transgenic line bearing *gcy-33* expression in the indicated pair of neurons, non-transgenic animals (-) and transgenic animals (+) are shown. Relative to non-transgenic controls (light gray bars), transgenic animals (dark gray bars) bearing *gcy-33* expression in BAG/URX or BAG only neurons restore body fat content to that seen in wild-type animals. Conversely, transgenic animals expressing *gcy-33* in only URX neurons fail to restore body fat relative to wild-type animals. Data are expressed as a percentage of body fat in wild-type animals + SEM (n = 18–20). NS, not significant and *, p<0.05 by one-way ANOVA including the Kruskal-Wallis correction. (E) Representative fluorescent image showing expression of *gcy-33*::*GFP* observed in BAG (closed arrowheads) and URX (open arrowheads) neurons. (F) Fat content was quantified in wild-type controls and worms with BAG neurons ablated. Data are expressed as a percentage of body fat in wild-type controls + SEM (n = 18–20). *, p<0.05 by Student’s t-test.

Because of our previously-described role for the URX-neuron-specific soluble guanylate cyclase GCY-36 in regulating oxygen-dependent fat loss [13], we examined all available null mutants of the soluble guanylate cyclase (sGC) family for changes in body fat (Figs 1A, S1A and S1B). Relative to wild-type animals *gcy-33* mutants showed a ~40–50% reduction in body fat stores, whereas other sGC loss-of-function mutants did not show an appreciable difference. Biochemical extraction of triglycerides from whole animals in wild-type and *gcy-33* mutants recapitulated this result (Fig 1B). Food intake (Fig 1C) and locomotor rates [17] are indistinguishable between wild-type and *gcy-33* mutants, suggesting that differences in the metabolism of fat stores underlies the fat phenotype of *gcy-33* mutants. *gcy-33* mutants are defective in the behavioral and neuronal responses to low oxygen, which are encoded by the BAG neurons [17, 19]. In addition to the BAG neurons, GCY-33 is also reported to be expressed in the URX neurons [17, 22]. To evaluate its site of action in regulating body fat, we restored *gcy-33* cDNA in the *gcy-33* mutants under its own promoter, and using heterologous promoters for the BAG and URX neurons. We observed robust partial rescue of body fat stores upon re-expression of GCY-33 under its endogenous promoter and in the BAG neurons, but did not observe meaningful rescue of body fat stores with its re-expression in the URX neurons (Fig 1D and 1E). Incomplete rescue of *gcy-33* mutants with exogenous cDNA has been previously observed for reasons that are currently not known [17]. Regardless, our experiments indicate that GCY-33 functions in the BAG neurons to regulate body fat stores. In accord with this hypothesis, we found that ablation of BAG neurons led to a profound decrease in body fat stores (Fig 1F).

### GCY-33 signaling from BAG neurons regulates oxygen-dependent fat loss via URX neuron signaling

**Fig 2:**
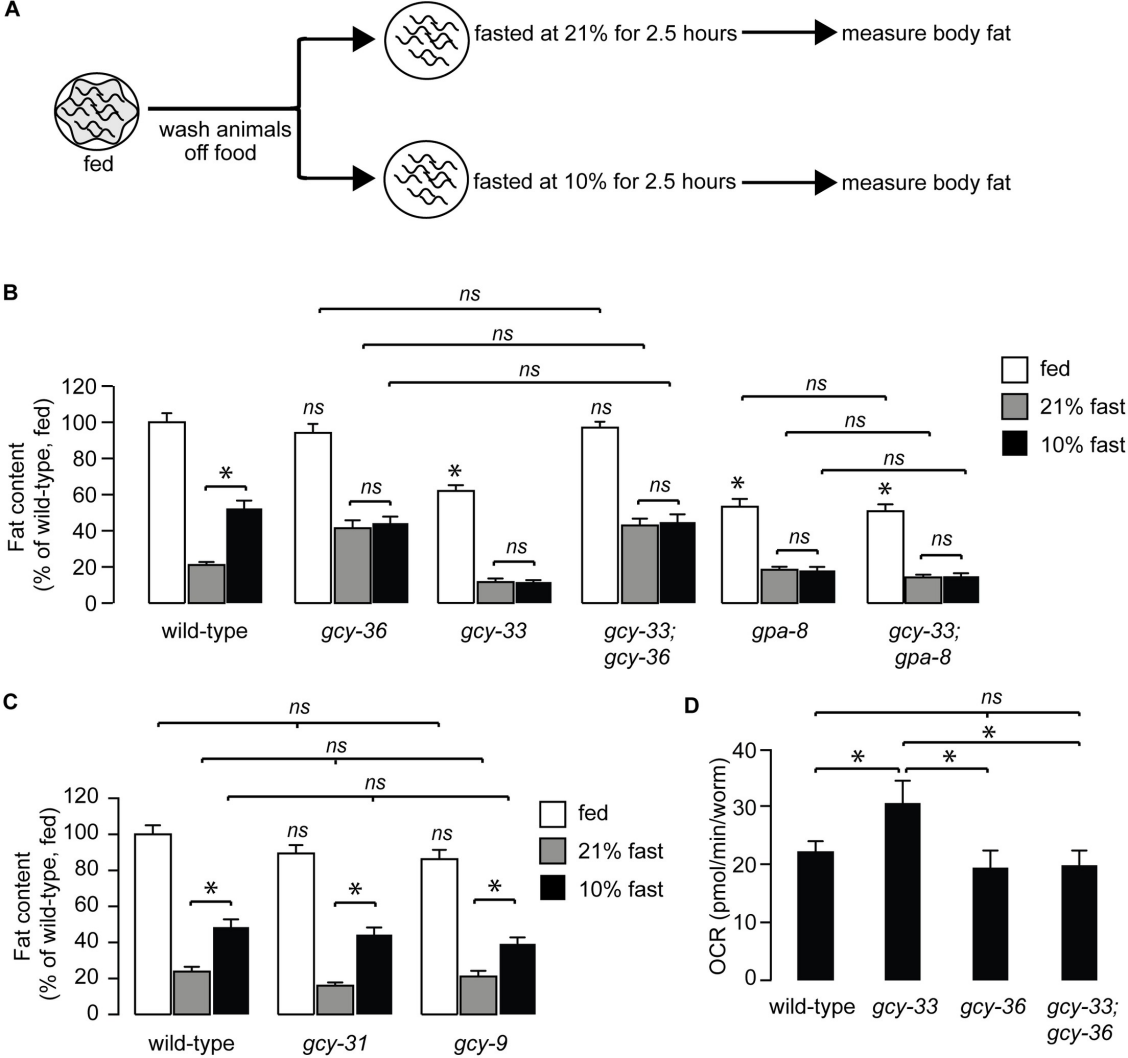
GCY-33 acts upstream of the URX-cGMP signaling pathway in fat regulation. (A) Schematic depiction of the oxygen-dependent fat loss assay. Worms were washed off food, and then fasted at either 21% (high) oxygen or 10% (low) oxygen. At the end of the fasting period, worms were fixed and stained with Oil Red O. (B) Worms of the indicated genotypes were subjected to the oxygen-dependent fat loss assay described in (A). White bars, fed; gray bars, fasted at 21% oxygen and black bars, fasted at 10% oxygen. Fat content was quantified for each genotype and condition. Data are expressed as a percentage of body fat in wild-type fed controls + SEM (n = 16–20). NS, not significant and *, p<0.05 by one-way ANOVA. (C) Worms of the indicated genotypes were subjected to the oxygen-dependent fat loss assay. Fat content was quantified for each genotype and condition. Data are expressed as a percentage of body fat in wild-type fed controls + SEM (n = 18–20). NS, not significant and *, p<0.05 by one-way ANOVA. (D) Oxygen consumption rate (OCR) in wild-type, *gcy-33*, *gcy-36*, and *gcy-33;gcy-36* mutants. Data are presented as pmol/min/worm + SEM (n = 5–15). *, p<0.05 by two-way ANOVA.

We wished to evaluate whether *gcy-33* mutants played a role in oxygen-dependent fat loss. Fasted wild-type adults initiate fat loss in an oxygen-dependent manner: wild-type animals exposed to 21% (henceforth, 'high') oxygen metabolize approximately twice as much body fat as those exposed to 10% (henceforth, 'low') oxygen (Fig 2A and 2B). We previously showed that this effect is not regulated by changes in food intake or locomotor rates at high versus low oxygen [13]. Rather, it occurs because of a selective metabolic shift towards fat utilization that is differentially regulated by oxygen availability. Thus, animals exposed to high oxygen show greater fat loss than those exposed to low oxygen for the same duration. As expected, *gcy-36* mutants show complete suppression of fat loss at high oxygen, consistent with the previously-observed role for the URX neurons in responses to high oxygen (Fig 2B). Interestingly, we found that *gcy-33* mutants showed the opposite phenotype to the *gcy-36* mutants: they had greater fat loss at low oxygen than wild-type animals, and were indistinguishable from those exposed to high oxygen. This result is consistent with loss of BAG neuron activity in *gcy-33* mutants [17], and indicates a role for BAG neurons in suppressing fat loss at low oxygen (Fig 2B). The observed effects of the BAG neurons on oxygen-dependent fat loss were specific to GCY-33 signaling, because null mutants of two other BAG-specific guanylate cyclases GCY-31 and GCY-9 [23] did not suppress oxygen-dependent fat loss (Fig 2C).

We were surprised to note that removal of the URX-specific sGC *gcy-36* fully suppressed the phenotype of *gcy-33* single mutants, such that the *gcy-33;gcy-36* mutants resembled the *gcy-36* single mutants alone (Fig 2B). Because GCY-36 functions in the URX neurons and not in the BAG neurons [17], this result suggested that the effect of GCY-33 signaling from the BAG neurons was dependent on GCY-36 signaling from the URX neurons (Fig 2B). In keeping with the increased fat loss, *gcy-33* mutants displayed increased energy expenditure that was fully suppressed by removal of *gcy-36* (Fig 2D).

The increased fat loss of *gcy-33* mutants under low oxygen (Fig 2B) was highly reminiscent of the *gpa-8* mutants which we had previously studied (Fig 2B; [13]). GPA-8 is a gustducin-like Gα protein which functions within the URX neurons themselves, and serves to inhibit GCY-36. *gpa-8* mutants have reduced fat stores in the fed state, show increased fat loss at low oxygen, and are indistinguishable from those fasted at high oxygen. *gcy-33;gpa-8* double mutants resembled each single mutant alone, with no additive effects (Fig 2B). We previously showed that the fat-regulatory property of *gpa-8* mutants arises from increased constitutive activation of the URX neurons because of GCY-36 de-repression [13], again resembling the *gcy-33;gcy-36* mutants. Together, the data suggests that GCY-33 signaling from BAG neurons negatively regulates URX neurons and oxygen-dependent fat loss.

### GCY-33 signaling and BAG neurons modulate the resting state of URX neurons

**Fig 3:**
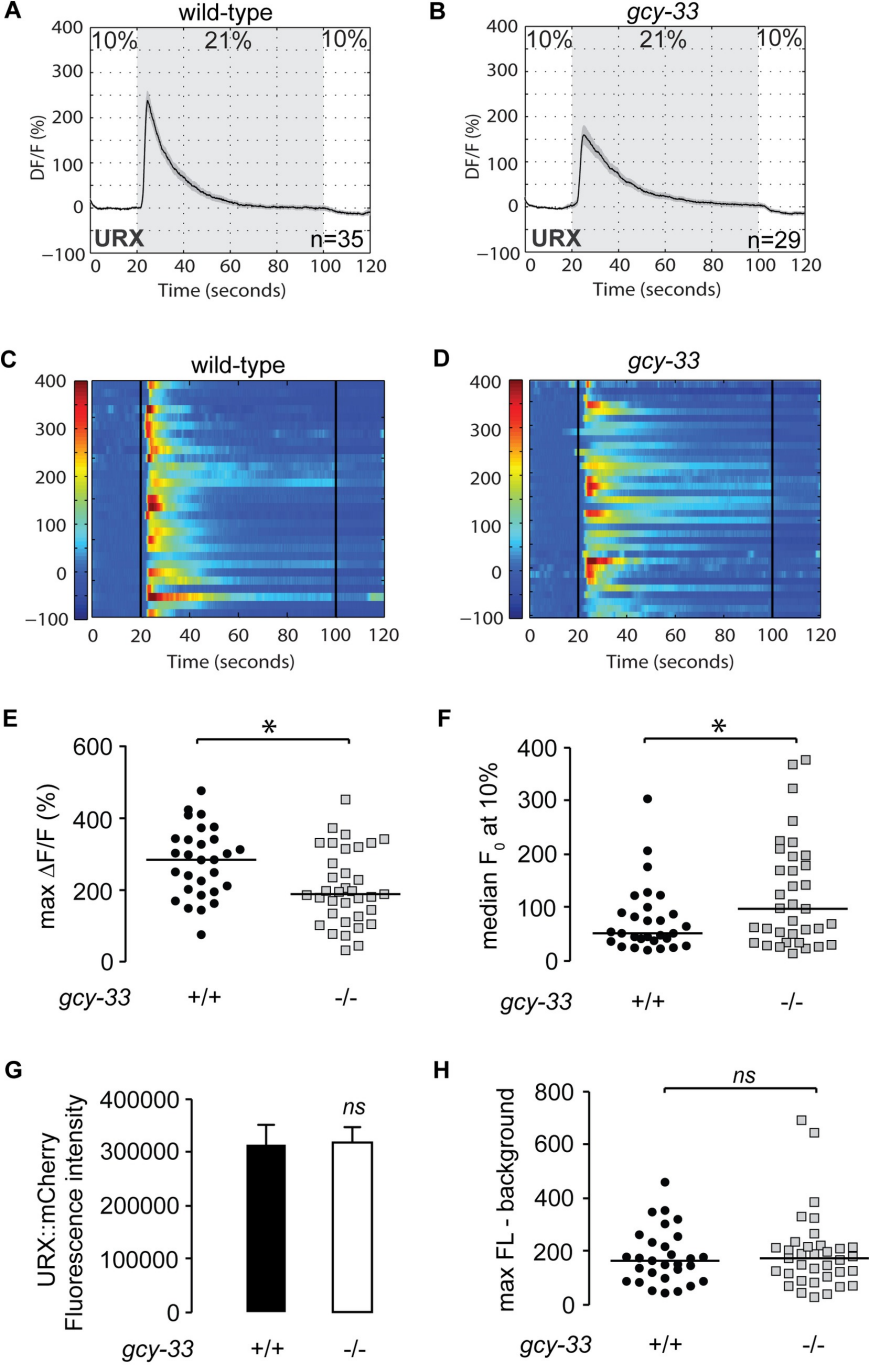
GCY-33 controls the resting-state Ca^2+^ concentrations in URX neurons. (A-D) Measurements of neuronal activity by Ca^2+^ imaging of URX neurons for each genotype. The number of animals used for each condition is shown in the figure. We conducted Ca^2+^ imaging experiments in URX neurons in living wild-type animals and *gcy-33* mutants bearing GCaMP5K under the control of the *flp-8* promoter. Oxygen concentrations in the microfluidic chamber were 10% (low) and 21% (high) as indicated. (A-B) For each genotype, black traces show the average percent change of GCaMP5K fluorescence (ΔF/F_0_) and gray shading indicates SEM. (C-D) Individual URX responses are shown for each genotype; each row represents one animal. (E) Maximum ΔF/F_0_ values are shown for individual wild-type animals and *gcy-33* mutants. Bars indicate the average value within each genotype. *, p<0.05 by Student’s t-test. (F) Individual baseline fluorescence (F_0_) values at 10% (low) oxygen are shown for individual wild-type animals and *gcy-33* mutants. Bars indicate the median value within each genotype. *, p<0.05 by Student’s t-test. (G) We imaged mCherry fluorescence in wild-type animals and *gcy-33* mutants expressing both GCaMP5K and mCherry under the control of the *flp-8* promoter. Images were taken in animals exposed to 10% (low) oxygen. For each genotype, the fluorescence intensity was imaged at the same exposure, determined to be within the linear range. Fluorescence intensity was quantified and expressed as an average + SEM (n = 20). NS, not significant by Student’s t-test. (H) The background-subtracted maximum fluorescence (max FL) at 21% (high) oxygen is shown for each wild-type animal and *gcy-33* mutant. Bars indicate the median value within each genotype. NS, not significant by Student’s t-test.

To directly study the effects of GCY-33 signaling on URX function, we measured neuronal activity using Ca^2+^ imaging in living animals. We used the genetically-encoded calcium indicator GCaMP5K as a reporter for URX activity which has been optimized for greater sensitivity to threshold activation properties [13, 24]. Wild-type animals bearing the GCaMP5K transgene expressed under the URX-specific promoter *flp-8* showed robust calcium influx at 21% oxygen (Fig 3A and 3C), as previously described [13, 25]. We observed two properties of URX activation in *gcy-33* mutants crossed into the *Pflp-8*::*GCaMP5K* transgenic line. First, at 21% oxygen there was an approximately 30% decrease in maximal activation of URX neurons in *gcy-33* mutants compared to wild-type animals (Fig 3B, 3D and 3E). Second, at 10% oxygen, we observed an approximately two-fold increase in baseline (F_0_) fluorescence values in *gcy-33* mutants compared to wild-type animals (Fig 3F). Importantly, we verified that there was no observed difference in *flp-8* promoter activity at 10% oxygen (Fig 3G). When measuring URX peak responses to the oxygen upshift in absolute GCaMP5K fluorescence levels, no significant difference between wild-type animals and *gcy-33* mutants was observed (Fig 3H). The major effect of GCY-33 therefore lies in controlling Ca^2+^ concentrations in the URX neurons at 10% oxygen. These data were highly reminiscent of the effect of *gpa-8* mutants on URX Ca^2+^ dynamics [13].

**Fig 4:**
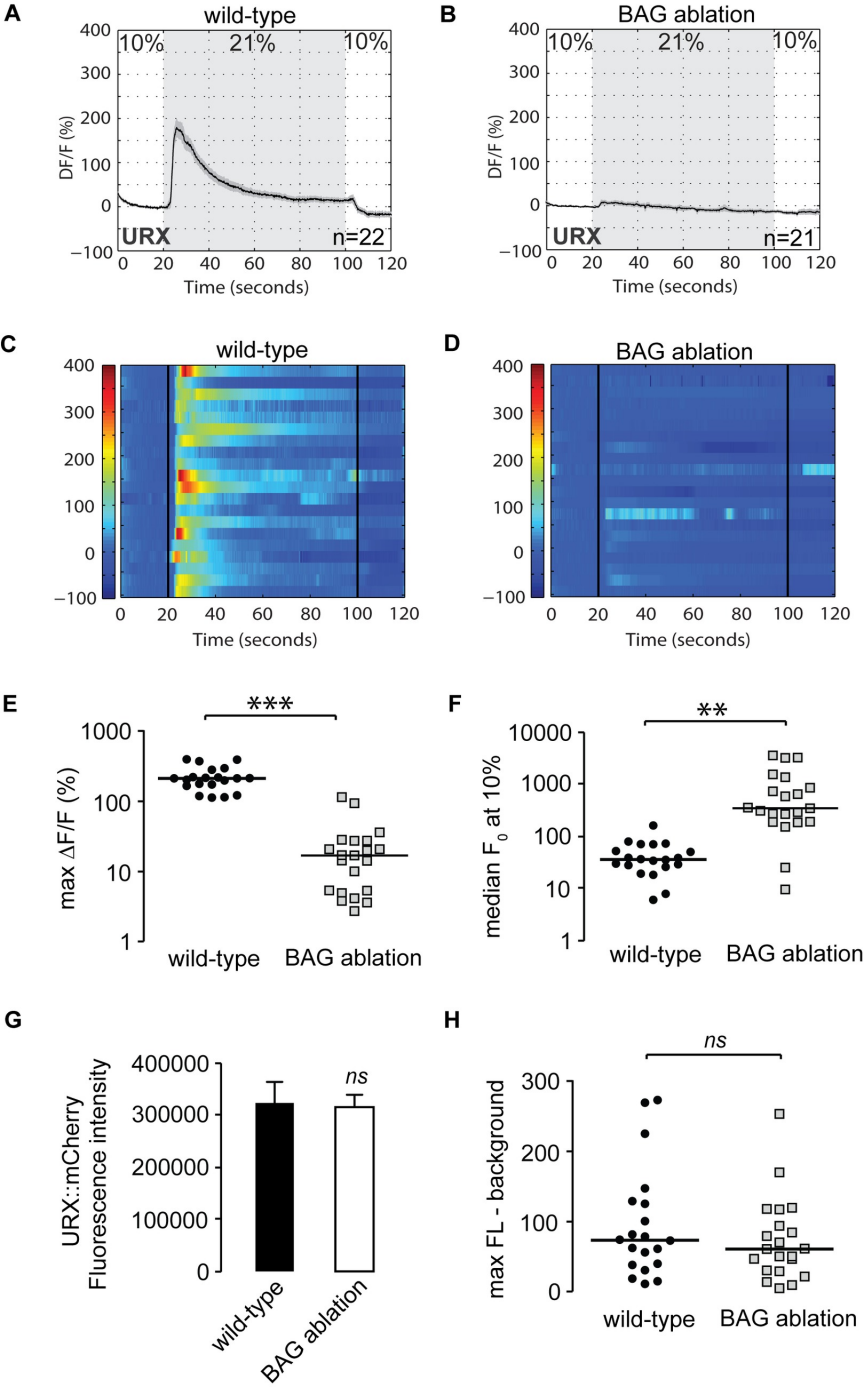
BAG neurons control the resting-state Ca^2+^ concentrations in URX neurons. (A-D) Measurements of neuronal activity by Ca^2+^ imaging of URX neurons for each genotype. The number of animals used for each condition is shown in the figure. We conducted Ca^2+^ imaging experiments in URX neurons in living wild-type and BAG-ablated animals bearing GCaMP5K under the control of the *flp-8* promoter. Oxygen concentrations in the microfluidic chamber were 10% (low) and 21% (high) as indicated. (A-B) For each genotype, black traces show the average percent change of GCaMP5K fluorescence (ΔF/F_0_) and gray shading indicates SEM. (C-D) Individual URX responses are shown for each genotype; each row represents one animal. (E) Maximum ΔF/F_0_ values are shown for individual wild-type and BAG ablated animals. Bars indicate the average value within each genotype. ***, p<0.001 by Student’s t-test. N.B. Log scale. (F) Individual baseline fluorescence (F_0_) values at 10% (low) oxygen are shown for individual wild-type animals and *gcy-33* mutants. Bars indicate the median value within each genotype. **, p<0.01 by Student’s t-test. N.B. Log scale. (G) We imaged mCherry fluorescence in wild-type and BAG ablated animals expressing both GCaMP5K and mCherry under the control of the *flp-8* promoter. Images were taken in animals exposed to 10% (low) oxygen. For each genotype, the fluorescence intensity was imaged at the same exposure, determined to be within the linear range. Fluorescence intensity was quantified and expressed as an average + SEM (n = 20). NS, not significant by Student’s t-test. (H) The background-subtracted maximum fluorescence (max FL) at 21% (high) oxygen is shown for each wild-type and BAG ablated animal. Bars indicate the median value within each genotype. NS, not significant by Student’s t-test.

We next decided to evaluate URX signaling properties in the absence of BAG neurons. To this end, we crossed the *Pflp-8*::*GCaMP5K* transgenic line with the BAG ablation transgenic line, and measured URX properties in the double transgenic animals. Remarkably, at 21% oxygen there was a complete absence of URX responses in animals lacking BAG neurons (Fig 4B, 4D and 4E). This effect occurred because of a 10-fold increase in median baseline (F0) values at 10% oxygen (Fig 4F). There were no changes in *flp-8* promoter activity or other observable features of the URX neurons, including URX peak responses (Fig 4G and 4H). Thus, BAG neurons, via *gcy-33* signaling, inhibit resting URX properties at low (10%) oxygen.

### Neuropeptide communication from BAG neurons to URX neurons coordinates oxygen-dependent fat loss

**Fig 5:**
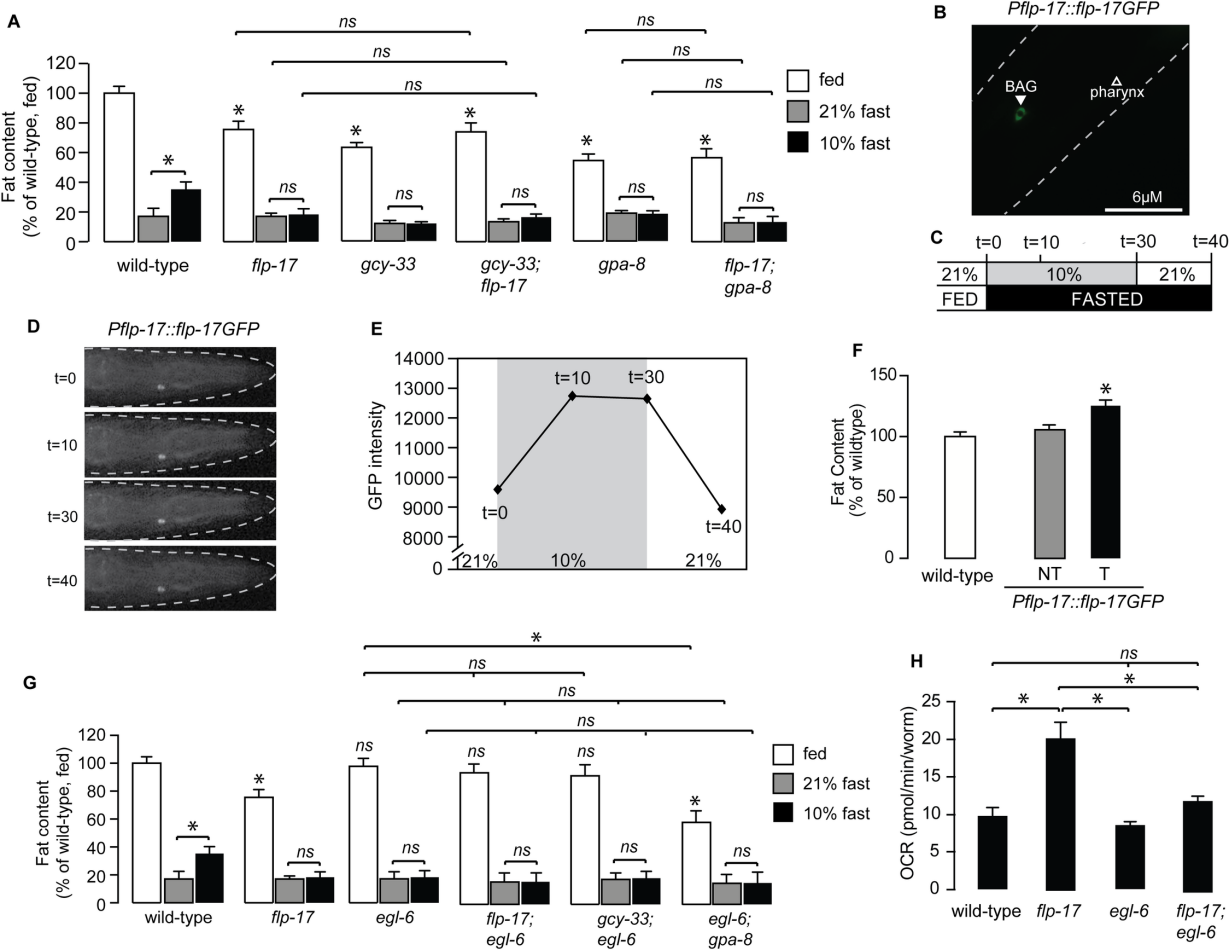
Neuropeptide communication from BAG neurons to URX neurons coordinates oxygen-dependent fat loss. (A) The indicated genotypes were subjected to the oxygen-dependent fat loss assay. Fat content was quantified for each genotype and condition. White bars, fed; gray bars, fasted at 21% oxygen and black bars, fasted at 10% oxygen. Data are expressed as a percentage of body fat in wild-type fed controls + SEM (n = 16–20). NS, not significant and *, p<0.05 by one-way ANOVA. (B) Representative image of a wild-type *C*. *elegans* expressing the *Pflp-17*::*flp17GFP* transgene in the BAG neurons. (C) Experimental paradigm to measure increase in FLP-17 secretion from BAG neurons, in response to oxygen switches. *Pflp-17*::*flp-17GFP* fluorescence was imaged immediately upon entering the chamber at 21% oxygen (t = 0 min). After the first image was taken, the oxygen concentration in the chamber was switched to 10%, and was imaged at 10 min (t = 10) and 30 min (t = 30). Immediately after the third image was taken, the oxygen concentration in the chamber was switched back to 21%, and *flp-17GFP* was imaged at 40 min (t = 40). (D) Representative images of data quantified in (E). n = 10, *, p<0.05 by one-way ANOVA. (F) flp-17 was overexpressed using the *Pflp-17*::*flp-17GFP* transgene in wild-type animals. Fat content was quantified for each genotype and condition. Relative to non-transgenic controls (gray bars), transgenic animals bearing the *flp-17* transgene under the control of its endogenous promoter had increased body fat content relative to non-transgenic controls (black bars). Data are expressed as a percentage of body fat in wild-type fed controls + SEM (n = 16–20). NS, not significant and *, p<0.05 by one-way ANOVA. (G) The indicated genotypes were subjected to the oxygen-dependent fat loss assay. Fat content was quantified for each genotype and condition. White bars, fed; gray bars, fasted at 21% oxygen and black bars, fasted at 10% oxygen. Data are expressed as a percentage of body fat in wild-type fed controls + SEM (n = 16–20). *, p<0.05 by one-way ANOVA.(H) Oxygen consumption rate (OCR) in wild-type, *flp-17*, *egl-6*, and *flp-17;egl-6* mutants. Data are presented as pmol/min/worm + SEM (n = 5–15). *, p<0.05 by two-way ANOVA.

BAG neurons are not known to form synaptic connections with the URX neurons. In addition, null mutants of the dense core vesicle-specific activator protein CAPS/UNC-31 that regulate neuropeptide secretion [26–28] blocked oxygen-dependent fat loss, whereas null mutants of the synaptic vesicle protein UNC-13 that regulates conventional neurotransmitter release [29, 30], did not (S2 Fig). To explore a potential neuropeptide-based mechanism of communication from the BAG neurons to the URX neurons, we conducted a genetic screen of all available mutants of the neuropeptide gene families (*flp*, *nlp* and *ins* gene families, 77/113 genes). Our top hit was a neuropeptide called FLP-17, the canonical BAG-neuron-specific neuropeptide [31–33] (Fig 5A). Interestingly, *flp-17* null mutants were nearly identical to the *gcy-33* mutants in oxygen-dependent fat loss. That is, relative to wild-type animals, fed *flp-17* mutants had reduced fat stores, showed increased fasting-dependent fat loss at low oxygen, and were indistinguishable from those at high oxygen. In addition, *gcy-33;flp-17* and *flp-17;gpa-8* double mutants resembled either single mutant alone (Fig 5A), suggesting that GCY-33 and FLP-17 from BAG neurons function in a linear pathway with GPA-8 signaling in URX neurons to inhibit fat loss at low oxygen.

We generated a *Pflp-17*::*flp-17GFP* transgenic line that showed robust and selective expression in the BAG neurons, as expected (Fig 5B). Interestingly, we observed punctate expression with GFP excluded from the nucleus, a pattern reminiscent of proteins destined for secretion [10]. To examine whether FLP-17 secretion is altered upon oxygen exposure, we measured FLP-17 secretion at low and high oxygen in these transgenic animals (Fig 5C). Interestingly, we observed that FLP-17 secretion increased at low oxygen, and returned to baseline at high oxygen (Fig 5C, 5D and 5E), in keeping with the activation of BAG neurons at low oxygen, and its inhibition at high oxygen [17]. Finally, we found that FLP-17 overexpression in BAG neurons significantly increased fat stores in the intestine (Fig 5F). Together, these data show that in response to low oxygen, BAG neurons control the resting state of URX neurons via the FLP-17 neuropeptide.

### The EGL-6 GPCR functions in URX neurons to regulate oxygen-dependent fat loss

A GPCR called EGL-6 functions as the cognate receptor for the FLP-17 neuropeptide in the control of egg-laying [33]. Activation of the EGL-6 receptor inhibits egg-laying via its function in the HSN neurons, whereas loss-of-function mutants do not have an egg-laying phenotype. We found that null mutants of the *egl-6* gene did not have overt defects in body fat stores, but were defective in oxygen-dependent fat loss indistinguishably from the *flp-17* mutants: loss of *egl-6* led to increased fat loss at low oxygen such that they resembled fat loss at high oxygen (Fig 5G). *flp-17;egl-6* double mutants resembled the *egl-6* single mutants in all respects: in the fed state they did not show an appreciable difference in body fat stores, and showed increased fat loss in low oxygen, without additive effects. *gcy-33;egl-6* and *egl-6;gpa-8* double mutants also did not yield additive effects relative to each single mutant alone, suggesting that these genes function in a linear pathway to regulate oxygen-dependent fat loss (Fig 5G). The decreased fat stores of *flp-17* mutants was accompanied by increased respiration (Fig 5H), whereas the *egl-6* null mutants showed no differences, as predicted by their body fat phenotype in the fed state (Fig 5G and 5H).

**Fig 6:**
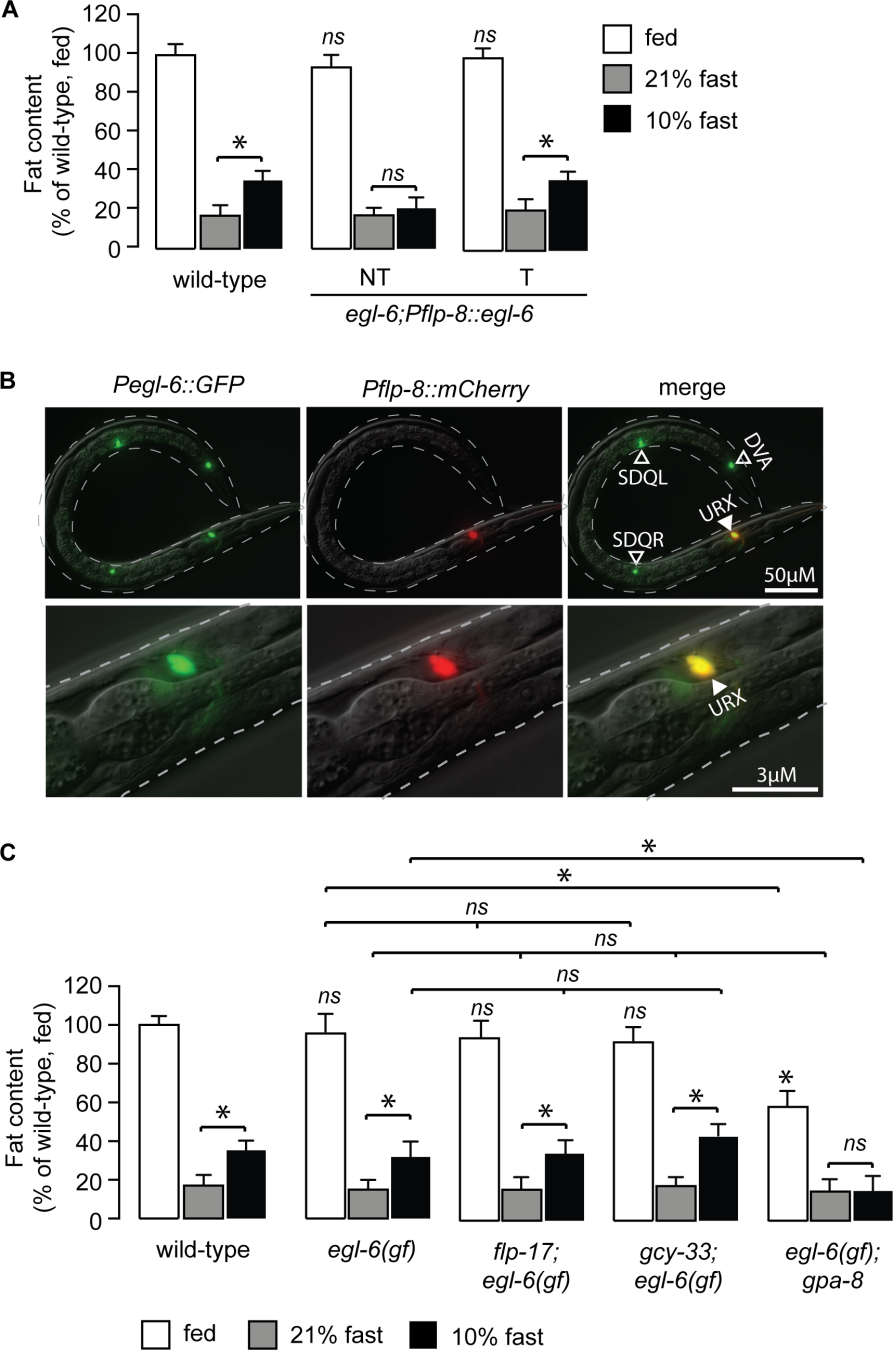
EGL-6 functions in URX neurons to regulate oxygen-dependent fat loss. (A) Worms from a transgenic line bearing *egl-6* expression using the *flp-8* promoter were subjected to the oxygen-dependent fat loss assay. Non-transgenic animals (-) and transgenic animals (+) are shown. Relative to non-transgenic controls (light gray bars), transgenic animals (dark gray bars) restore body fat content at 10% (low) oxygen to that seen in wild-type animals. Data are expressed as a percentage of body fat in wild-type animals + SEM (n = 16–20). NS, not significant and *, p<0.05 by one-way ANOVA. (B) Representative fluorescent images showing the expression of P*egl-6*::*GFP* (left panels), P*flp-8*::*mCherry* (central panels), and their co-localization in URX (merge, right panels). *egl-6* expression was also observed in DVA, SDQL, SDQR, HSN (not depicted) and additional head neurons. (C) The indicated genotypes were subjected to the oxygen-dependent fat loss assay. Fat content was quantified for each genotype and condition. White bars, fed; gray bars, fasted at 21% oxygen and black bars, fasted at 10% oxygen. Data are expressed as a percentage of body fat in wild-type fed controls + SEM (n = 16–20). NS, not significant and *, p<0.05 by one-way ANOVA.

Because our signaling pathway indicated communication from BAG neurons to URX neurons via the FLP-17 neuropeptide, we tested whether EGL-6 functions in the URX neurons. In *egl-6* null mutants, we restored expression in the URX neurons and measured oxygen-dependent fat loss. Relative to non-transgenic animals, *egl-6* re-expression in URX neurons fully restored oxygen-dependent fat loss (Fig 6A), suggesting that EGL-6 functions in the URX neurons to limit fat loss at low oxygen. *egl-6* expression had been noted in *C*. *elegans* head neurons [33], and we observed clear and robust expression of *egl-6* in the URX neurons (Fig 6B). The *egl-6* gain-of-function allele, *n592*, displayed oxygen-dependent fat loss indistinguishably from wild-type animals, and completely suppressed the oxygen-dependent fat loss phenotype of *flp-17* and *gcy-33* mutants, but not of the *gpa-8* mutants (Fig 6C). Together, these data provide compelling evidence for a signaling pathway from the BAG neurons to the URX neurons for oxygen-dependent fat loss, via neuropeptide communication.

### The BAG neuropeptide FLP-17 controls URX baseline responses

**Fig 7:**
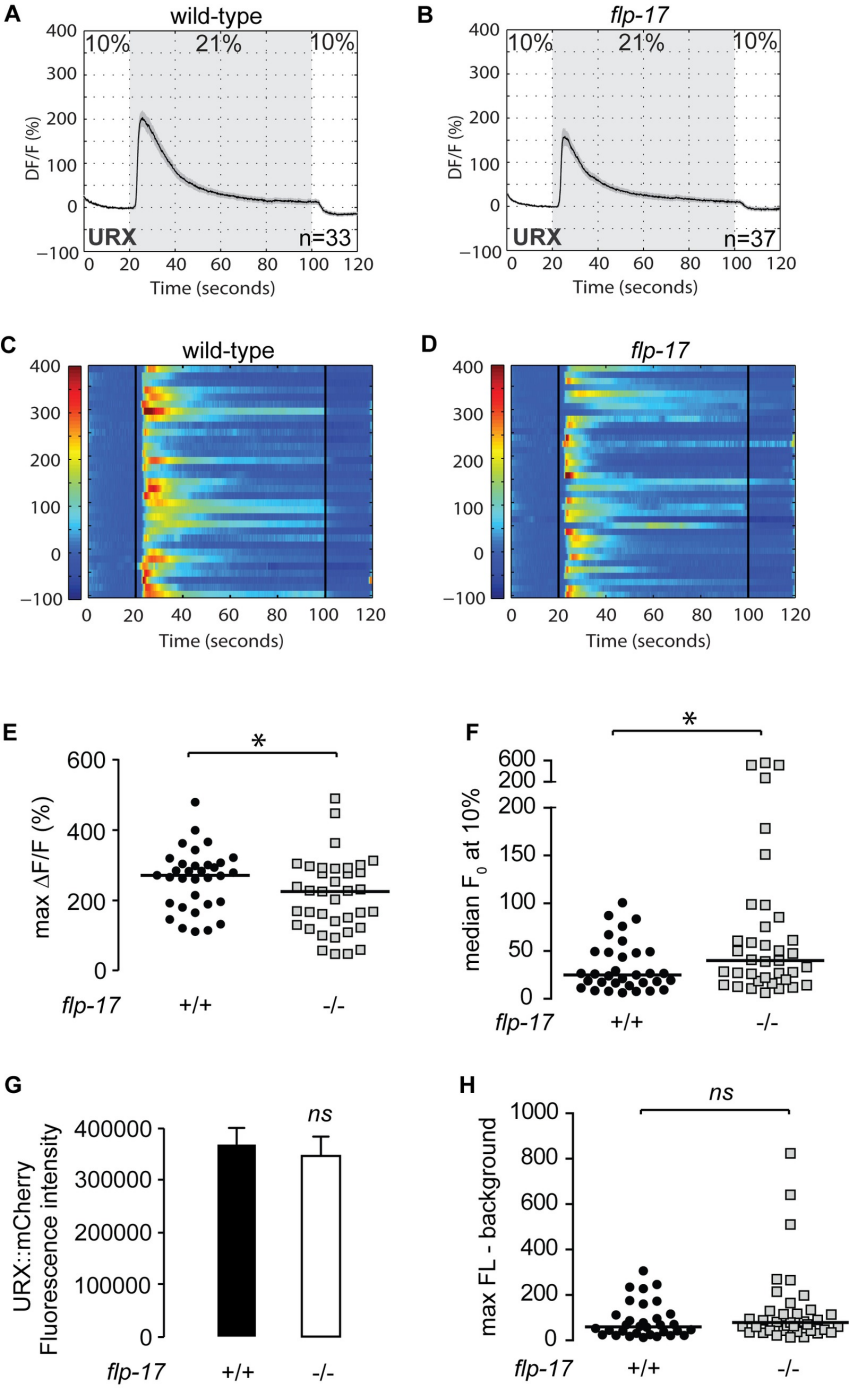
The BAG neuropeptide FLP-17 controls URX baseline responses. (A-D) Measurements of neuronal activity by Ca^2+^ imaging of URX neurons for each genotype. The number of animals used for each condition is shown in the figure. We conducted Ca^2+^ imaging experiments in URX neurons in living wild-type animals and *flp-17* mutants bearing GCaMP5K under the control of the *flp-8* promoter. Oxygen concentrations in the microfluidic chamber were 10% (low) and 21% (high) as indicated. (A-B) For each genotype, black traces show the average percent change of GCaMP5K fluorescence (ΔF/F_0_) and gray shading indicates SEM. (C-D) Individual URX responses are shown for each genotype; each row represents one animal. (E) Maximum ΔF/F_0_ values are shown for individual wild-type animals and *flp-17* mutants. Bars indicate the average value within each genotype. *, p<0.05 by Student’s t-test. (F) Individual baseline fluorescence (F_0_) values at 10% (low) oxygen are shown for individual wild-type animals and *flp-17* mutants. Bars indicate the median value within each genotype. *, p<0.05 by Student’s t-test. (G) We imaged mCherry fluorescence in wild-type animals and *flp-17* mutants expressing both GCaMP5K and mCherry under the control of the *flp-8* promoter. Images were taken in animals exposed to 10% (low) oxygen. For each genotype, the fluorescence intensity was imaged at the same exposure, determined to be within the linear range. Fluorescence intensity was quantified and expressed as an average + SEM (n = 20). NS, not significant by Student’s t-test. (H) The background-subtracted maximum fluorescence (max FL) at 21% (high) oxygen is shown for each wild-type animal and *flp-17* mutant. Bars indicate the median value within each genotype. NS, not significant by Student’s t-test.

**Fig 8:**
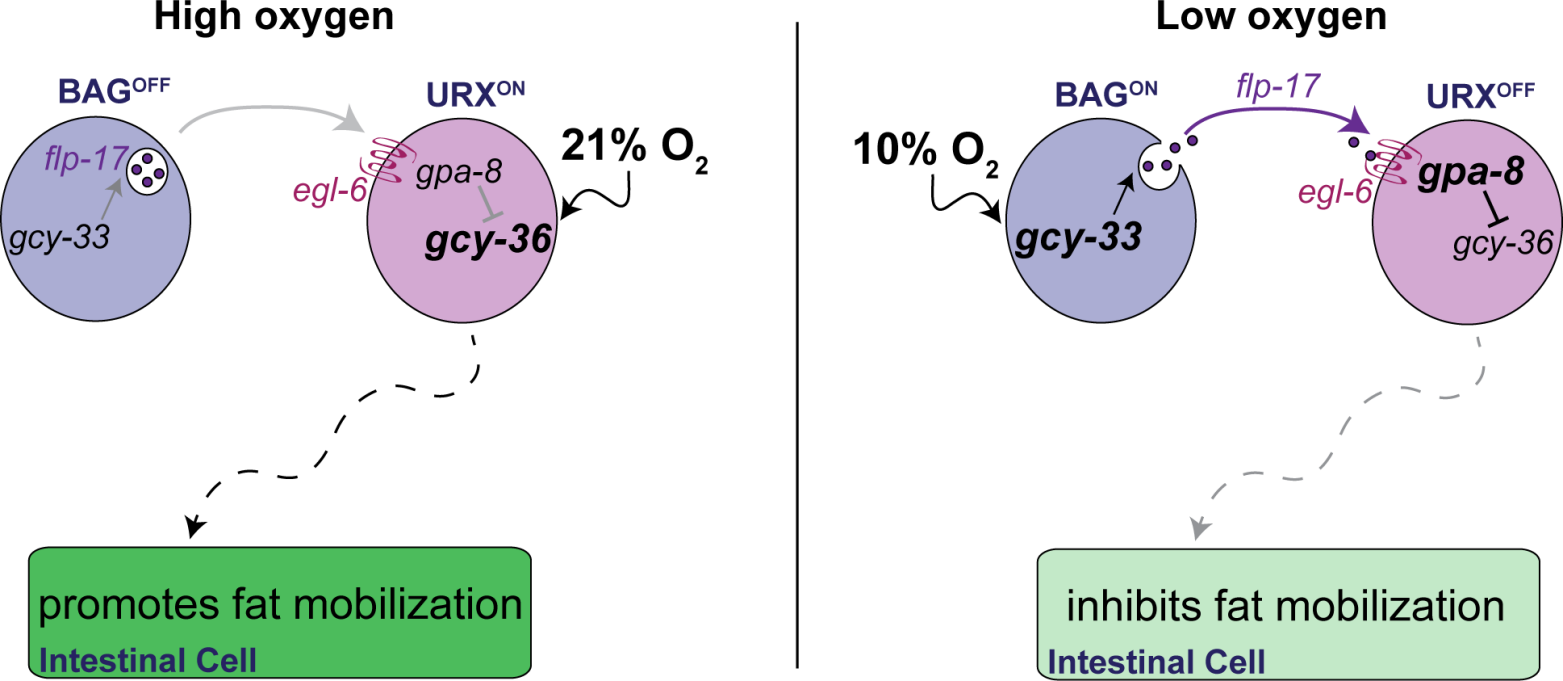
Model depicting the role of the oxygen-sensing neurons in intestinal lipid metabolism. When animals are exposed to high (21%) oxygen (left panel), the URX neurons are activated via the soluble guanylate cyclase GCY-36, which promotes fat loss in the intestine. Under low (10%) oxygen (right panel), signaling via GCY-33 in the BAG neurons promotes the release of the neuropeptide FLP-17, which is detected by the EGL-6 GPCR in the URX neurons. Communication from BAG neurons to the URX neurons, mediated by FLP-17/EGL-6 signaling dampens URX neuron activity, thus inhibiting fat loss. Our model provides a mechanism by which tonic activity of the URX neurons is controlled, and the symbiotic relationship between the BAG neurons and the URX neurons that converts the perception of oxygen to tune the rate and extent of fat loss in the intestine.

For additional verification of the role of FLP-17 in controlling URX functions, we measured URX responses in the absence of *flp-17*. Wild-type animals bearing the GCaMP5K transgene expressed under the URX-specific promoter *flp-8* showed robust calcium influx at 21% oxygen (Figs 7A and 3C), as previously described [13, 25]. We observed two properties of URX activation in *flp-17* mutants crossed into the *Pflp-8*::*GCaMP5K* transgenic line. First, at 21% oxygen there was an approximately 25% decrease in maximal activation of URX neurons in *flp-17* mutants compared to wild-type animals (Fig 7B, 7D and 7E). Second, at 10% oxygen, we observed an approximately 1.5-fold increase in baseline (F_0_) fluorescence values in *flp-17* mutants compared to wild-type animals (Fig 7F). Importantly, we verified that there was no observed difference in *flp-8* promoter activity at 10% oxygen (Fig 7G). When measuring URX peak responses to the oxygen upshift in absolute GCaMP5K fluorescence levels, no significant difference between wild-type animals and *flp-17* mutants was observed (Fig 7H). The major effect of FLP-17 therefore lies in controlling Ca^2+^ concentrations in the URX neurons at 10% oxygen. These data were highly reminiscent of the effect of *gcy-33* and *gpa-8* mutants on URX Ca^2+^ dynamics [13]. Taken together, our results describe a neuropeptide-based signaling pathway from BAG to URX neurons that regulates the resting properties of URX neurons, and show that resting state modulation can have a major and lasting impact on metabolic homeostasis (Fig 8).

## Discussion

In this report, we show that the *C*. *elegans* oxygen-sensing neurons reciprocally regulate peripheral lipid metabolism. Under lower oxygen conditions, the BAG sensory neurons respond by repressing the URX neurons, which are tonic sensors of higher oxygen. Molecularly, repression occurs via a neuropeptide secreted from the BAG neurons, and its cognate receptor that functions in the URX neurons (Fig 8). Thus, BAG sensory neurons counterbalance the metabolic effect of the tonically active URX neurons. The physiological consequence of this repression is to limit fat utilization when oxygen levels are low.

In previous work we had defined the role of the URX neurons in stimulating fat loss in response to high environmental oxygen (Fig 8, left panel). When oxygen levels are high and food supplies dwindle, URX neurons are activated via GCY-36, ultimately leading to increased fat loss via the ATGL-1 lipase [13]. It has been noted that high oxygen coincides with dwindling bacteria, the main food source for *C*. *elegans*, because decreases in bacterial respiration would drop the local ambient concentration of oxygen below atmospheric (21%). In the present study we define a mechanism by which the URX neurons are held in the 'off state' under conditions of low oxygen, that is, via repression from BAG neurons. It has been appreciated for some years that URX neurons are tonic sensors of environmental oxygen [17], suggesting that they must be turned off by an active repression mechanism, the molecular basis of which remained unknown. Our model suggests that BAG neurons in *C*. *elegans* play an essential role in mediating tonic repression of URX-neuron resting properties in the following way: the sensation of low oxygen concentrations by the guanylate cyclase GCY-33 in the BAG neurons initiates the release of the neuropeptide, FLP-17, which is detected via the GPCR EGL-6 in the URX neurons. FLP-17/EGL-6 signaling leads to decreased basal activity of URX neurons via the GPA-8-mediated repression of GCY-36. Thus, in low oxygen, URX neurons are retained in the 'off state', ultimately limiting fat loss (Fig 8, right panel). Collectively, URX neurons are held in the off state in two ways. First, by internal sensing of body fat stores [13] and second, by the presence of food or low oxygen via the BAG neurons (this study). We suggest that it is a combination of decreased GCY-33 activation in BAG neurons and increased GCY-36 activation in URX neurons that tunes the rate and extent of fat utilization in the intestinal cells.

Work presented here uncovers the molecular basis for the conversion of oxygen sensation via the chemosensory system, to the regulation of lipid metabolism. Although the molecular nature of oxygen sensors in mammals is not yet known, oxygen concentration in blood, cerebrospinal fluid and other organs of the body vary widely [34–37]. We speculate that mammalian chemosensors of oxygen would similarly play a role in regulating lipid homeostasis in a manner that is independent of hypoxia-sensing [37]. The role of tonic repression is another interesting facet of sensory control that has emerged from our studies. Although tonic repression of neural activity is not well understood, our data indicate that neuropeptide-mediated repression of resting state is a robust mechanism to control the activity of sensory neurons. We suggest that tonic repression via neuropeptides and systemic hormones offers a broad and powerful mechanism to relay state information and control sensory and metabolic responses across the organs of the body.

Our data indicate that the stimulation of fat loss is far more complex than what can be elicited simply by reduction in food intake or increased locomotion. Rather, environmental conditions, relayed by sensory systems, tune the rate and extent of fat loss which is in turn governed by complex genetic interactions between the nervous system and the intestine. We suggest that the mammalian counterparts to such regulatory pathways will be broadly informative for our understanding of fat metabolism.

## Materials and methods

### Animal maintenance and strains

*C*. *elegans* was cultured as described [38]. N2 Bristol, obtained from the Caenorhabditis Genetic Center (CGC) was used as the wild-type reference strain. The mutant and transgenic strains used are listed in S1 Table. Animals were synchronized for experiments by hypochlorite treatment, after which hatched L1 larvae were seeded on plates with the appropriate bacteria. All experiments were performed on day 1 adults.

### Cloning and transgenic strain construction

Promoters and genes were generated using standard PCR techniques from N2 Bristol worm lysates or cDNA and cloned using Gateway Technology^TM^ (Life Technologies). Promoter lengths were determined based on functional rescue and are outlined in S2 Table. All rescue plasmids were generated using polycistronic GFP. Transgenics were constructed by microinjection into the *C*. *elegans* germline followed by visual selection of transgenic animals under fluorescence. For the microinjections, 5–25 ng/μl of the desired plasmid was injected with 25 ng/μl of a *Punc-122*::*GFP* or *Pmyo-3*::*mCherry* coinjection marker and 50–70 ng/μl of an empty vector to maintain a total injection mix concentration of 100 ng/μl. In each case, 10–20 stable transgenic lines were generated. Two lines were selected for experimentation based on consistency of expression and transmission rate. For GCaMP5K transgenic animals, 5 ng/μl of *Pflp-8*::*GCaMP5K* was injected with 2 ng/μl of a *Pflp-8*::*mCherry* coinjection marker.

### Oil Red O staining

Oil Red O staining was performed as described [13]. Within a single experiment, roughly 3,500 animals were fixed and stained, 100 animals were visually inspected on slides, following which 15–20 animals were imaged for each genotype/condition. All experiments were repeated at least 3 times.

### Image acquisition and quantitation

Black and white images of Oil Red O stained animals and fluorescent images were captured using a 10X objective on a Zeiss Axio Imager microscope. Lipid droplet staining in the first four pairs of intestinal cells was quantified by measuring background-subtracted pixel intensity after setting a standard threshold. Within each experiment, approximately 15–20 animals at the same stage of adulthood were quantified from each condition without selection bias. Images were quantified using ImageJ software (NIH).

### Lipid extraction and quantification

For each group, 2,000 worms/10 cm plate were grown for 44 h at 25°C. After washing with PBS twice, worms were flash frozen in liquid nitrogen. Worms were homogenized in PBS containing 5% TritonX100 and proteinase inhibitor (Thermo Scientific), and lipid was extracted using the TissueLyser II (QIAGEN). Triglyceride content was measured using the EnzyChrom Triglyceride Assay Kit (BioAssay Systems), and triglyceride levels were normalized to total protein, which was determined using the Pierce BCA Protein Assay (Thermo Scientific).

### Food intake

Food intake was measured by counting pharyngeal pumping, as previously described [39]. For each animal, the rhythmic contractions of the pharyngeal bulb were counted over a 10 s period under a Zeiss M2 Bio Discovery microscope. For each genotype, 10 animals were counted per condition and the experiment was repeated at least three times.

### Oxygen-dependent fat loss assay

The experiments were conducted as described [13]. For each strain, approximately 3,500 synchronized L1 larvae were seeded onto each of three plates. Worms were grown at 20°C for 48 h after which all plates were transferred to the bench top. Worms subjected to the fasting protocol were washed off the plates with PBS in 5 sequential washes over a 30-minute period to eliminate residual bacteria, and then seeded onto NGM plates without food. Worms were then subjected to a 2.5 h fasting period at either 21% or 10% oxygen. To establish the time course of fasting, pilot experiments were conducted at atmospheric (21%) oxygen. The “21% fasted” plates were placed in a non-airtight container at room temperature. The “fed” control plates were placed in a similar but separate container. The “10% fasted” plates were placed in a custom-designed sealed acrylic oxygen chamber (TSRI Instrumentation and Design Lab), fitted with inlet and outlet valves. The inlet valve was connected via bubble tubing to a pressurized oxygen and nitrogen pre-mixture containing 10% oxygen (Praxair, Inc.), and the outlet valve was exposed to air. All plates were positioned right side up without lids. The sealed chamber was then perfused for 15 min with 10% oxygen. Following perfusion, both valves were closed. During the experiment, pressure inside the chamber was held constant, as judged by a gauge placed inside the oxygen chamber. The chamber was kept at room temperature for an additional 2.25 h, so that all fasted conditions remained off food for a total of 2.5 h following the washes. At the end of this period, worms from the respective conditions were collected for Oil Red O staining.

### Oxygen consumption

Oxygen consumption rates (OCR) were recorded using the Seahorse XFe96 Analyzer (Agilent). Worms were grown at 15°C for 24 h and then moved to 20°C for 48 h. Worms were washed with M9 buffer and transferred into a well in a 96-well plate at approximately 10 worms per well. The final volume per well was 200 μL of M9. Worms were without food for 60 min prior to the first OCR recording. Respiration was measured according to the manufacturer's instructions. Oxygen consumption rates were normalized to worms per well. In all genotypes tested, we did not observe any changes in worm size, growth or developmental stage.

### Calcium imaging

We used a microfluidic chamber constructed with the oxygen-permeable poly(dimethylsiloxane) (PDMS) as described [17]. A Valvebank II (AutoMate Scientific, Inc.) was used to control input from two pressurized pre-mixtures of oxygen and nitrogen containing either 10% oxygen or 21% oxygen (Praxair, Inc.). The gas flow rate was set to 0.26 psi at the outlet of the chamber as judged by a VWR^TM^ traceable pressure meter. Immediately before imaging, individual day 1 adult animals were sequentially transferred to two unseeded plates. Individual *C*. *elegans* adults were then transported into the chamber in a drop of S Basal buffer containing 6mM levamisole (Acros Organics B.V.B.A.) via Tygon tubing (Norton). Animals were constantly submerged in S Basal buffer while inside the chamber. After the animals were immobilized inside the chamber, GCaMP5K fluorescence was visualized at 40x magnification using a spinning disk confocal microscope (Olympus) using MetaMorph^TM^ (version 6.3r7, Molecular Devices). Worms were pre-exposed to 10% oxygen for 5 min in the microfluidic chamber as described [17]. GCaMP5K fluorescence was recorded by stream acquisition for 2 min at a rate of 8.34 frames/second, with an exposure time of 20 ms using a 12-bit Hamamatsu ORCA-ER digital camera. Each animal was recorded once. GCaMP5K-expressing neurons were marked by a region of interest (ROI). The position of the ROI was tracked using the “Track Objects” function in MetaMorph^TM^. An adjacent ROI was used to subtract background from the total integrated fluorescence intensity of the ROI. Data were analyzed using MATLAB (MathWorks, Inc.). Fluorescence intensity is presented as the percent change in fluorescence relative to the baseline (ΔF/F_0_). F_0_ was measured in worms exposed to 10% oxygen during the first 9–13 seconds for each recording and calculated as an average over that period. All animals were day 1 adults at the time of imaging. The number of animals used for each condition is denoted in the figures.

### Oxygen dependent FLP-17 secretion

We used a microfluidic chamber constructed with the oxygen-permeable poly(dimethylsiloxane) (PDMS) as described [17]. A Valvebank II (AutoMate Scientific, Inc.) was used to control input from two pressurized pre-mixtures of oxygen and nitrogen containing either 10% oxygen or 21% oxygen (Praxair, Inc.). The gas flow rate was set to 0.26 psi at the outlet of the chamber as judged by a VWR^TM^ traceable pressure meter. Immediately before imaging, individual day 1 adult animals were sequentially transferred to two unseeded plates. Individual *C*. *elegans* adults were then transported into the chamber in a drop of S Basal buffer containing 6mM levamisole (Acros Organics B.V.B.A.) via Tygon tubing (Norton). *Pflp-17*::*flp-17GFP* animals were constantly submerged in S Basal buffer while inside the chamber, with the oxygen concentration set to 21%. After the animals were immobilized inside the chamber, fluorescence was imaged at 40x magnification using a spinning disk confocal microscope (Olympus) using MetaMorph^TM^ (version 6.3r7, Molecular Devices) (t = 0). Immediately after the first image was taken, the oxygen concentration in the chamber was changed to 10%. With the worm exposed to 10% oxygen, fluorescence was imaged again after 10 min (t = 10) and 30 min (t = 30). Immediately after the third image was taken, the oxygen concentration in the chamber was changed to 21%. After 10 min at 21% oxygen, fluorescence was imaged again (t = 40). Images were quantified using ImageJ software (NIH).

### Statistics

Wild-type animals were included as controls for every experiment. Error bars represent SEM. Student’s t-test, one-way ANOVA, and two-way ANOVA were used as indicated in the figure legends.

## Supporting information

S1 Fig *gcy-33* null mutants exhibit a significant decrease in body fat content. (A) Images of wild-type animals and *gcy-33* mutants fixed and stained with Oil Red O. Animals are oriented facing upwards with the pharynx at the top of each image. For each genotype, images depict the full range of the observed phenotype. (B) The integrated density of the lipid droplets is used to quantify body fat stores, as described in the Materials and Methods. Graph represents the integrated density values of individual wild-type animals and *gcy-33* mutants. *, p<0.05 by Student’s t-test.
(TIF)

S2 Fig Neuropeptidergic signaling drives fat mobilization through oxygen sensing. Worms of the indicated genotypes were subjected to the oxygen-dependent fat loss assay. Fat content was quantified for each genotype and condition. Data are expressed as a percentage of body fat in wild-type fed controls + SEM (n = 20). NS, not significant and *, p<0.05 by one-way ANOVA.
(TIF)

S1 Table *C*. *elegans* strains used in this study. (TIF)

S2 Table Promoters used in this study. (TIF)
