# Supplementary figures and images for: Oxygen-sensing neurons reciprocally regulate peripheral lipid metabolism via neuropeptide signaling in Caenorhabditis elegans
Source: PLoS Genet. 2018 Mar 26;14(3):e1007305. doi: 10.1371/journal.pgen.1007305 (PMC5886693; doi:10.1371/journal.pgen.1007305)

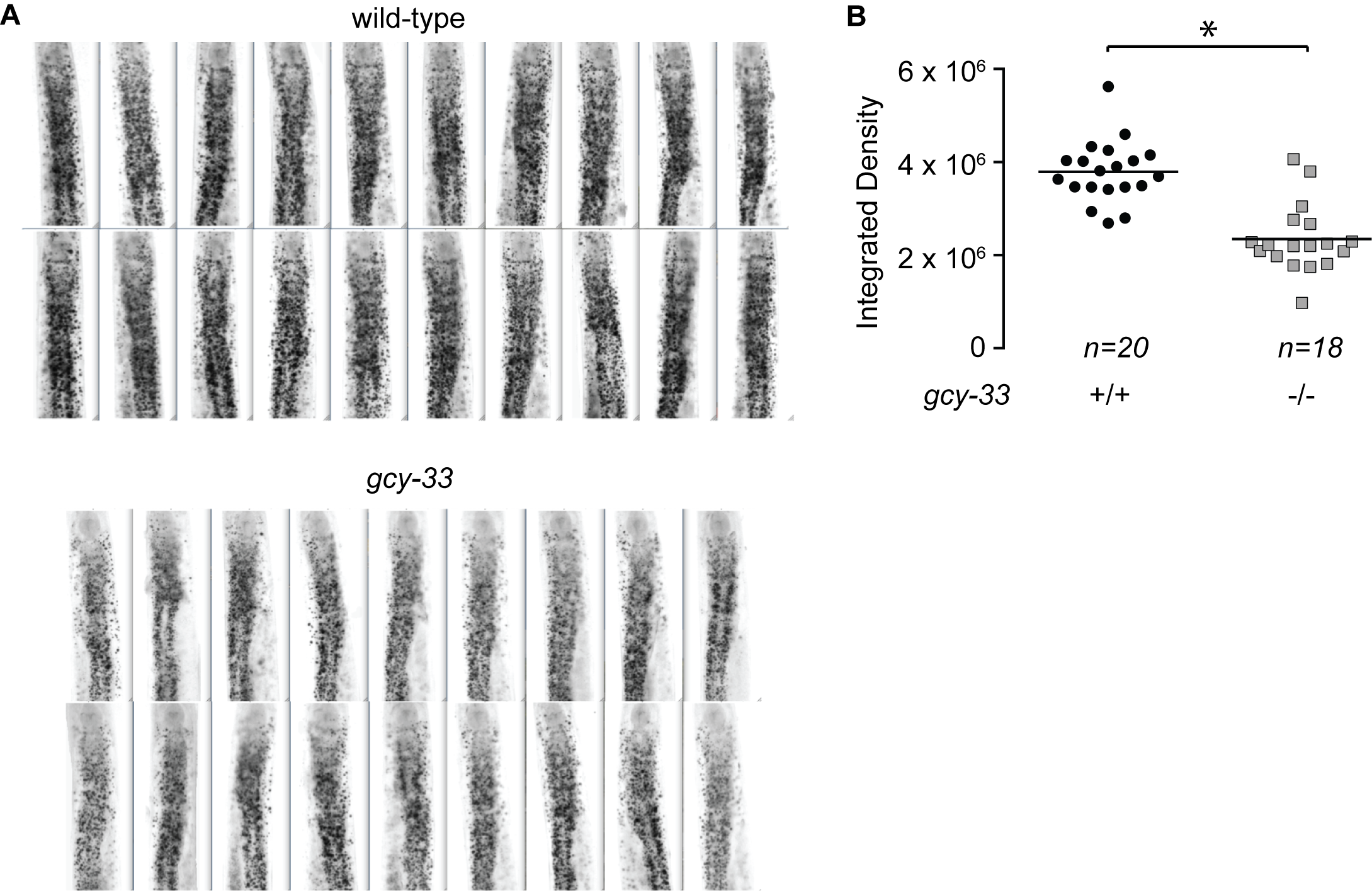

Supplement: S1 Fig — (A) Images of wild-type animals and gcy-33 mutants fixed and stained with Oil Red O. Animals are oriented facing upwards with the pharynx at the top of each image. For each genotype, images depict the full range of the observed phenotype. (B) The integrated density of the lipid droplets is used to quantify body fat stores, as described in the Materials and Methods. Graph represents the integrated density values of individual wild-type animals and gcy-33 mutants. *, p<0.05 by Student’s t-test. (TIF) [file pgen.1007305.s001.tif]

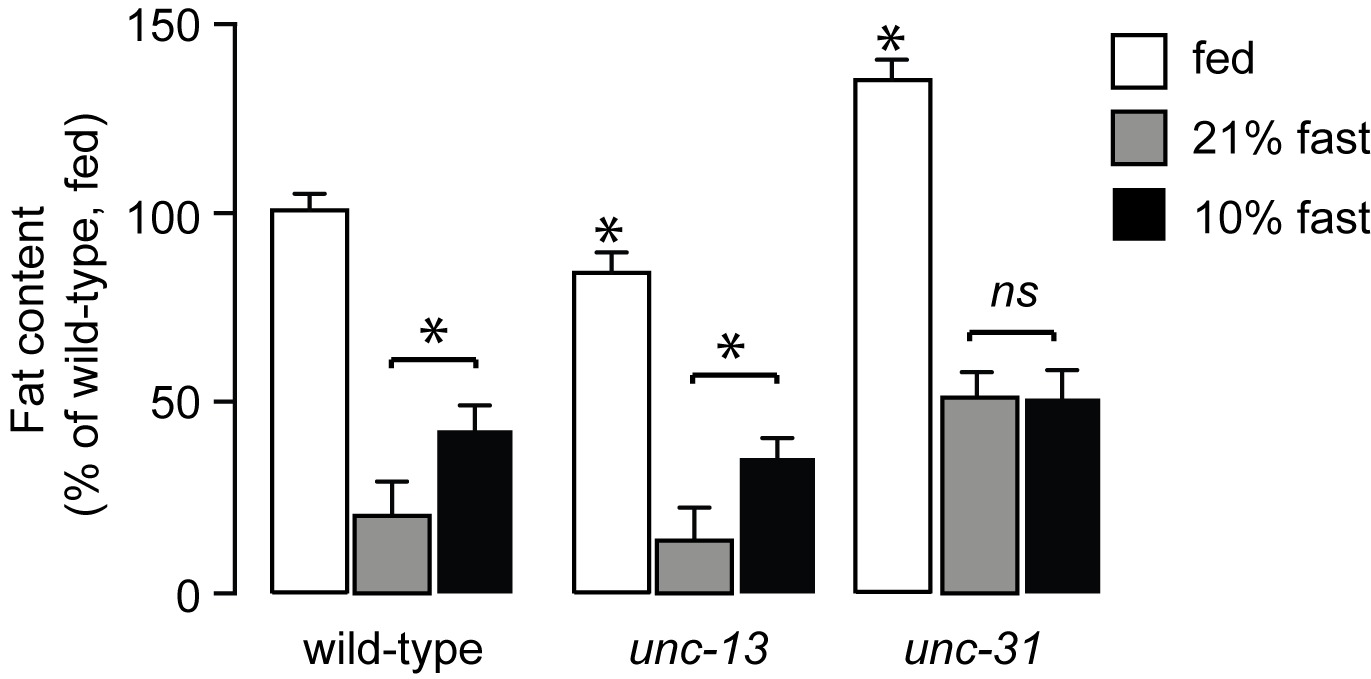

Supplement: S2 Fig — Worms of the indicated genotypes were subjected to the oxygen-dependent fat loss assay. Fat content was quantified for each genotype and condition. Data are expressed as a percentage of body fat in wild-type fed controls + SEM (n = 20). NS, not significant and *, p<0.05 by one-way ANOVA. (TIF) [file pgen.1007305.s002.tif]
